# Preliminary in-silico analysis of vascular graft implantation configuration and surface modification

**DOI:** 10.1038/s41598-023-42998-y

**Published:** 2023-10-02

**Authors:** Ray Prather, Yashar Pourmoghadam, Joseph Fadhli, Faris Al-Mousily, Kamal Pourmoghadam

**Affiliations:** 1 Department of Biomedical Sciences, University of Central Florida, Orlando, FL USA; 2 Department of Molecular and Cell Biology, University of California Berkeley, Berkeley, CA USA; 3 Department of Medicine, College of Medicine, University of Central Florida, Orlando, FL USA; 4 Department of Surgery, College of Medicine, University of Central Florida, Orlando, FL USA

**Keywords:** Biotechnology, Computational biology and bioinformatics, Cardiology, Medical research, Engineering

## Abstract

Vascular grafts are used to reconstruct congenital cardiac anomalies, redirect flow, and offer vascular access. Donor tissue, synthetic, or more recently tissue-engineered vascular grafts each carry limitations spanning compatibility, availability, durability and cost. Synthetic and tissue-engineered grafts offer the advantage of design optimization using in-silico or in-vitro modeling techniques. We focus on an in-silico parametric study to evaluate implantation configuration alternatives and surface finishing impact of a novel silicon-lined vascular graft. The model consists of a synthetic 3D-generic model of a graft connecting the internal carotid artery to the jugular vein. The flow is assumed unsteady, incompressible, and blood is modeled as a non-Newtonian fluid. A comparison of detached eddy turbulence and laminar modeling to determine the required accuracy needed found mild differences mainly dictated by the roughness level. The conduit walls are modeled as non-compliant and fixed. The shunt configurations considered, are straight and curved with varied surface roughness. Following a grid convergence study, two shunt configurations are analyzed to better understand flow distribution, peak shear locations, stagnation regions and eddy formation. The curved shunt was found to have lower peak and mean wall-shear stress, while resulting in lower flow power system and decreased power loss across the graft. The curved smooth surface shunt shows lower peak and mean wall-shear stress and lower power loss when compared to the straight shunt.

## Introduction

Implantation of a vascular graft to bypass blood flow is a common surgical technique used to redirect flow around an obstructed area, reconstruct congenital cardiac anomalies, repair an injured vessel or provide vascular access. The length, diameter and the configuration of the conduit may vary depending on the type of reconstruction and the size of the patient. For hemodialysis, a vascular access can be created by anastomosing an artery and a nearby vein to form an arteriovenous fistula. This technique requires native in-situ vascular mobilization to create the arteriovenous connection. In coronary bypass surgery either a native arterial in-situ, venous, or arterial interposition graft of varying length is implanted to improve myocardial perfusion to the region of an obstructed coronary artery. Vascular grafts can also be used for complex vascular reconstructions in remodeling of blood flow in congenital heart defects. For example, in the three-stage palliative procedures to reconstruct single ventricle type congenital heart defects, a Blalock–Thomas–Taussig Shunt (BTTS) is temporarily created to provide pulmonary blood flow from the systemic circulation. Despite wide spread use of bypass grafts, several limitations exist, including the availability of in-situ vessel when harvesting from a patient, donor compatibility and durability^1^. Synthetic grafts such as polytetrafluoroethylene (PTFE) have long been used as alternatives, however; long-term patency and thrombogenicity are of increasing concern^2^. Recently tissue engineered vascular graft (TEVG) have gained prominence as viable alternatives, addressing issues of compatibility, availability and potential thrombogenicity. However, studies have shown poor histological assessment of these grafts with luminal stenosis observed at 4 weeks and intima-media thickening observed at 6 weeks after surgery^3^. Past studies have used in-vivo animal models to characterize patency rates in ovine and swine models with PTFE arteriovenous grafts between the carotid artery (CA) and the jugular vein (JV). The findings showed that thrombosis caused failure in 18% of PTFE grafts within 1 week^4, 5^ and significantly reduced patency 8 weeks post-implantation^6^. Similarly, in a swine model, implanted PTFE grafts in the iliac artery resulted in stenosis in 80% of the cases within 6–8 weeks^7^. In yet another study, over half of PTFE grafts connecting the femoral artery to the femoral vein showed hyperplasia 2 weeks post-surgery at graft-vein anastomosis^8^. A CA-JV TEVG obtained from decellularized arterial scaffolds seeded with ovine endothelium proved structurally sound but became stenosed 4 months following implantation^9^. Alternative scaffolds for TEVG using small intestinal sub-mucosa (SIS) for developing new vascular grafts have been proposed. A key factor is the similarity to native vascular tissue, since the SIS is primarily comprised of collagen, elastin, fibronectin, and other factors^10–13^. Vascular SIS grafts have already been successfully tested in an in-vivo swine model as an abdominal aorta implant^12, 13^. These grafts were also compared to standard GORE-TEX® grafts in sheep and swine models. While both implants maintained patency for over a month, the C-shaped SIS grafts showed significantly more prolonged patency, neovascularization and regeneration^14^. Overall, the previous evaluations of arteriovenous shunt morphology, geometry and size in the form of computational and clinical analyses have been inconclusive in showing a superior design for optimizing flow pattern and durability^15–17^.

In this study, the aim is to use in-silico modeling to accurately track the flow field resulting from two proposed graft implantation configurations to evaluate the viability of the proposed surgical approach that will then lead to more detailed optimization studies targeting shunt surface finish for novel silicon grafts to minimize platelet activation, thrombus formation and neointimal growth.

## Methods

### Geometry model

**Figure 1:**
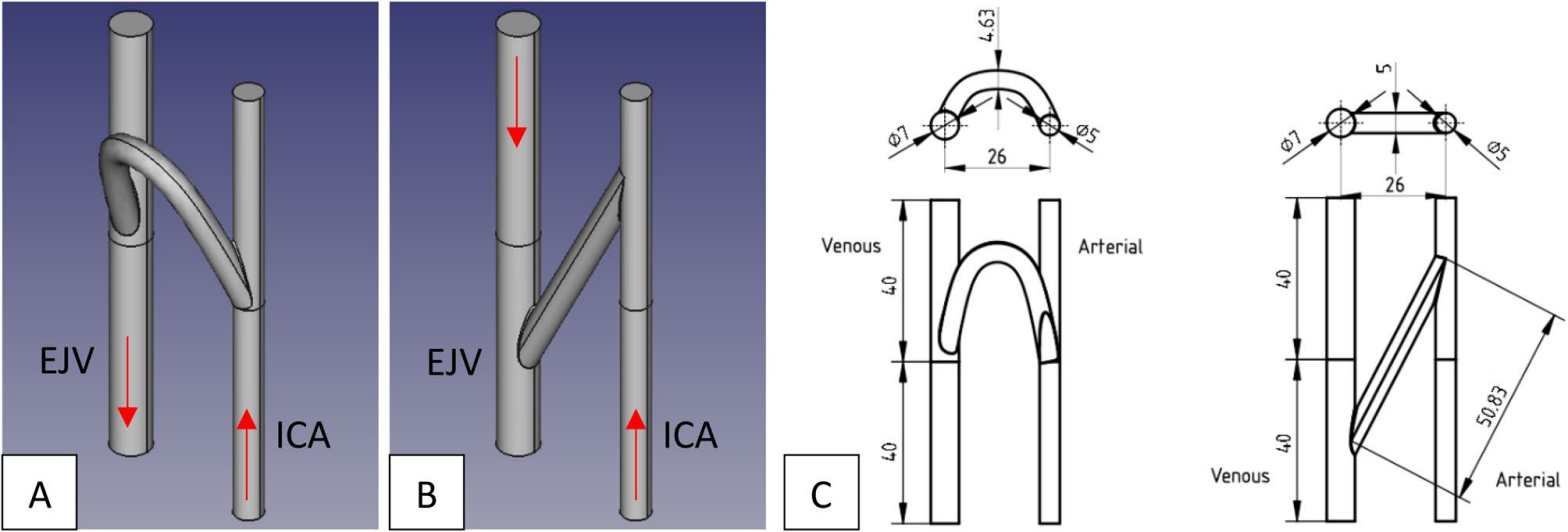
FreeCAD rendered geometries for (**A**) curved graft (C-shunt or CS) and (**B**) straight graft (S-shunt or SS) implanted between internal carotid artery (ICA) and internal jugular vein (EJV). The red arrows indicate blood flow direction. (**C**) Schematic for both graft configurations displaying conduit sizes in (mm).

For this preliminary study the model under consideration will be simplified to test the proposed graft implantation configurations in the region of interest. The shunt placements preview a C-shape (C-shunt or CS) implantation with an out of plane bend (Fig. 1A,C) and an in-plane shallow-angle backward straight shunt (S-shunt or SS) connection (Fig. 1B,C). Based on in-vivo animal and human studies documented in literature and data provided by our clinical partners, a generic synthetic model of a vascular graft connecting the internal carotid artery (ICA) to the external jugular vein (EJV) was modeled using the open-source CAD software FreeCAD (https://www.freecadweb.org). The use of EJV is due to it being the dominant vein in sheep which is the animal model of choice for future in-vivo studies. Once the modeling was completed, a STL file was generated and imported in the destination software StarCCM + (CD-Adapco, Siemens), where the geometry can then be partitioned in the various inlet, outlet, and wall boundaries. The shunt was specifically designed to have a diameter of 5 mm and a length of about 5 cm.

The fluid domain mesh was directly generated in Star-CCM+, using an unstructured polyhedral mesh including up to 10 wall-boundary prism layers with a thickness at maximum of 1 base size and slow surface growth rate generating upwards of 1.1–1.4 M cells (supplementary material Fig. S1). Unstructured polyhedral mesh elements are well suited for complex geometrical topologies resulting in high quality grids and retaining stability (and improved gradient calculations) given the increased number of shared faces with neighboring cells^18, 19^. Studies have shown polyhedral meshes improving convergence properties for less dense meshes (~ 60% less iterations with ~ 77% fewer cells) as compared to tetrahedral meshes^19^. High curvature refinement was also implemented to ensure a proper wall boundary mesh near the graft anastomosis locations. A mesh independence study was carried out and resulted in meshes with maximum base size of 0.5 mm and refinement up to 0.06 mm (near wall, anastomosis locations and curvature)^20, 21^.

### Computation fluid dynamics

The generic model is imported into Star-CCM+, which is a commercial multi-physics finite volume based computational fluid dynamics (CFD) solver developed by CD-Adapco and currently maintained by Siemens. The fluid domain is modeled as an incompressible (constant density), non-Newtonian fluid with blood density $\rho$ of 1060 kg/m^3^ and non-linear viscosity is implemented using a 3-parameter Carreau–Yasuda model (Eq. 1) based on clinical data for 40% hematocrit where ${\mu }_{\infty }$ is the infinite-shear dynamic viscosity, ${\mu }_{o}$ is the zero-shear dynamic viscosity, $\dot{\gamma }$ is the shear rate, and λ is the relaxation-time constant^22^. This model allows us to alter blood properties to account for fluctuations in hematocrit levels or to simulate anticoagulants being administered. The flow field solution is obtained by solving the mass conservation (Eq. 2) and the Navier–Stokes (Eq. 3) equations.

1 $$\left(\dot{\gamma }\right)={\mu }_{\infty }+\left({\mu }_{o}-{\mu }_{\infty }\right)\frac{1}{{\left[1+{\left(\uplambda \dot{\gamma }\right)}^{2}\right]}^\frac{1}{3}}$$

2 $$\nabla \cdot \overrightarrow{V}=0$$

3 $$\rho \frac{\partial \overrightarrow{V}}{\partial t}+\rho \left(\nabla \cdot \overrightarrow{V}\right)\overrightarrow{V}=-\nabla p+\nabla \cdot \sigma \; {\text{where}} \; \sigma =\mu \left(\dot{\gamma }\right)\left[\nabla \overrightarrow{V}+{\left(\nabla \overrightarrow{V}\right)}^{T}\right]$$

In this study Star-CCM+ solves these equations with an unsteady implicit solver with a second order time discretization and bounded-central convective scheme. Convergence criteria for normalized residuals at each time-step was set to < 0.001. To determine the flow regime in the fluid domain the Reynolds number was estimated first by volume-averaging flow velocity across the domain and then surface-averaging at four distinct cross-sections (proximally and distally on both arterial and venous sides). The first estimation finds flow to be in the laminar regime with *Re* ≈ 1100–1750. However, upon closer inspection it was found that in some regions flow may become turbulent due to higher flow velocity with the *Re* ≈ 2000–3300. In order to model complex patterns in the field, flow is modeled as turbulent using an Elliptic Blending (EB) *κ*–*ε* Detached Eddy Simulation (DES), combining features of Reynolds–Averaged Navier–Stokes (RANS) for irrotational and near-wall regions, and Large Eddy Simulation (LES) in unsteady separated regions. In Star-CCM+ the EB-DES is implemented as a Delayed Detached Eddy Simulation (DDES) that improves the distinction between regions employing RANS or LES despite grid refinement. This modeling approach allows surface roughness to be accounted by wall roughness models as opposed to an impractical mesh refinement. In the current implementation, roughness maintains physical meaning as long as the non-dimensional roughness remains smaller than the wall-adjacent cell height (Eq. 4):

4 $${R}^{+}=\frac{r\rho {u}^{*}}{\mu }<{y}^{+}$$

where ${R}^{+}$ is the non-dimensional surface roughness, $r$ is the roughness, ${u}^{*}$ is the velocity scale, and ${y}^{+}$ is the cell height. To ensure the grid resolution is sufficient to capture the desired flow features, the modeled and resolved turbulent kinetic energies we measured following a grid convergence study. This analysis confirmed that in turbulent regions the flow is largely resolved (ratio of resolved flow > 60%).

The patient under consideration is an adult with a cardiac output (CO) of 5 L/min at rest. The boundary conditions (or BC) at all the inlets (proximal ICA and proximal EJV) are given as a mass flow rate inlets in units of [kg/s]. We assume that the ICA provides 5% of the total CO, and the EJV inlet flow is 10% of the CO. At the wall boundary a no-slip condition (zero velocity) is applied. The BCs at the outlets (distal ICA and distal EJV) are given as pressures in units of [Pa]. ICA outlet pressure is set at 70 mmHg, and the EJV outlet pressure is 5 mmHg. For simplicity the current model assumes the wall boundary as fixed and non-compliant, hence no dynamic fluid–body interaction or fluid–structure interaction modeling has been included.

### Disclosure

This study did not involve any animal trials. No patient data was used in this study.

## Results

### Mesh independence study

**Figure 2:**
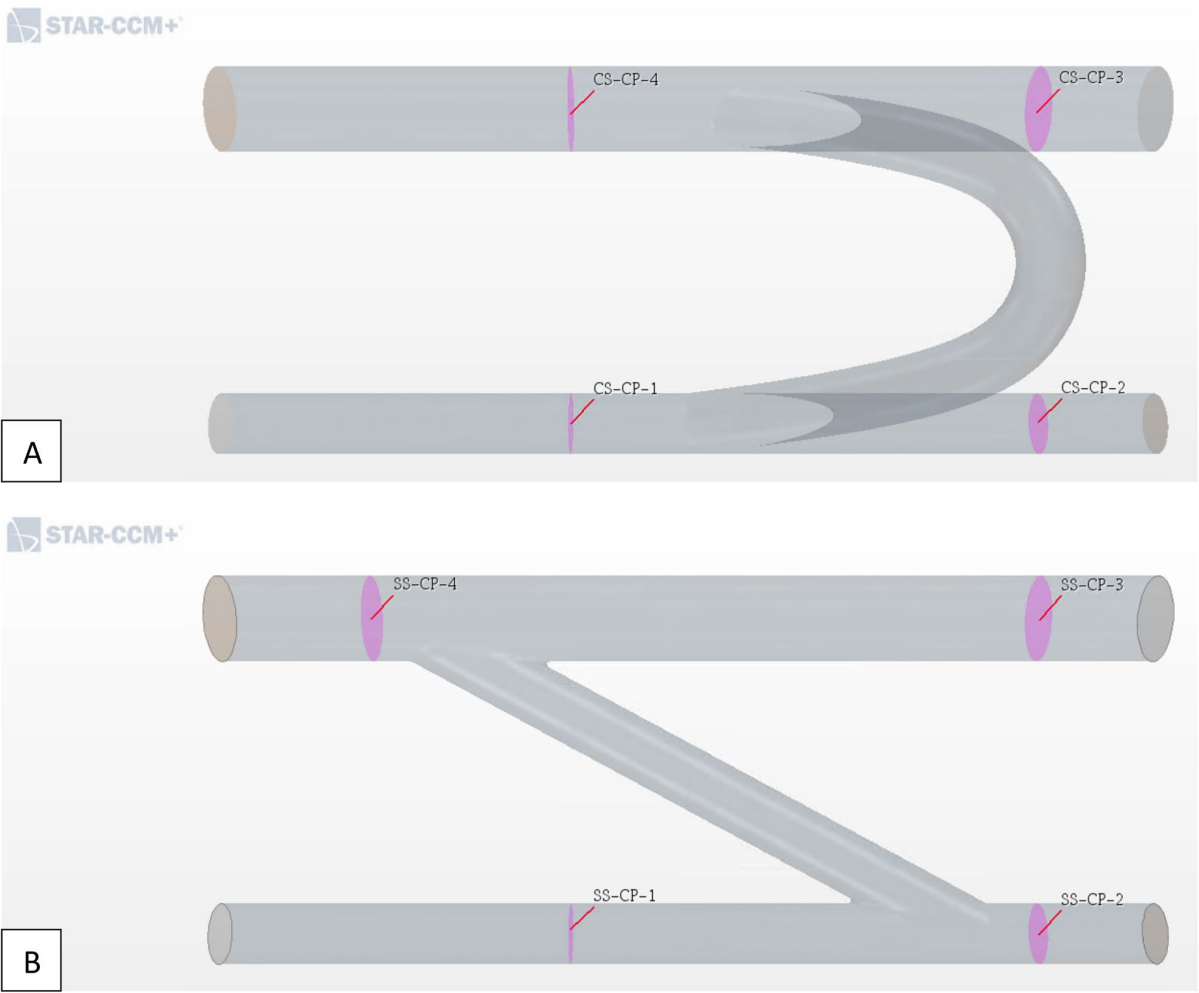
Quantification of flow velocity and pressure sampled at 4 locations (CP-1 = arterial proximal, CP-2 = arterial distal, CP-3 = venous proximal, CP-4 = venous distal) for grid independence study, proximal and distal to the graft anastomosis locations on both arterial and venous sides. This analysis was carried out for both models, (**A**) C-shape (CS) and (**B**) straight (SS) grafts (*CP* constraint planes).

We tracked velocity, static pressure, and wall shear stress as markers to indicate a grid converged flow solution for both models. Data for pressure and velocity were surface-averaged at four different cross-sectional planes (constraint planes or CP), proximal and distal to the graft anastomosis locations (Fig. 2). Wall shear stress was averaged over the whole domain wall surface. Tables S1 and S2 in the supplementary materials provide a detailed summary of the grid independence study for mesh base size ranging 0.5–2.0 mm, tracking 3 hemodynamic quantities. Roache’s grid convergence index (GCI), order of convergence and asymptotic range of convergence are reported.

A cursory comparison for increasing mesh refinement between cases 1–5 reveals good convergence for each flow field variable. Calculation of the rate of convergence (ROC) based on the GCI for the three most dense meshes (GCI_34_ and GCI_45_) confirms our previous observation for asymptotic grid convergence (i.e., $ROC\approx 1.00$), hence the domain will implement an unstructured polyhedral mesh featuring a base size of 0.5 mm, high curvature refinement and 10 prism layers. For three velocity quantities (CS-V-1 and SS-V-1) it was not possible to report the GCI and ROC due to non-monotonic convergence (i.e. the solution oscillates about a value), however it can be observed that these values are nearly constant.

### Flow field

To ensure that the results obtained following the implementation of the EB *κ*–*ε* DES turbulence model are meaningful the roughness parameter ${R}^{+}$ was compared to the cell height ${y}^{+}$. As alluded in the methods section, physically meaningful results are obtained when ${R}^{+}<{y}^{+}$. Table S3 in the supplementary materials shows that the converged mesh respects the expected proportionality for both models and each roughness considered in this study.

To inspect the resultant flow field and verify the effectiveness of the shunt implantation, the field is qualified with contour pressure plots along the main geometrical plane and flow patterns are tracked by means of velocity streamlines. This ensures both arterial and venous pressure maintain appropriate pressure ranges dictated by the BC, while the streamlines monitor velocity ranges across the fluid domain and compare them to expected values. A wall shear stress distribution plot along the wall allows for further scrutiny in peak shear location that should be found near the graft anastomosis. In addition, the volume flow rate, static pressure, average velocity, and wall shear stress are collected in Table 3 at the same sample cross-sectional planes used for the grid convergence study (Fig. 2).

Tables 1A–C summarize flow field data for volume flow rate (VF), pressure (P), velocity (V), and surface averaged wall shear stress, proximal and distal to the graft anastomosis location on both arterial and venous sides, flow modeling and surface roughness. Pressure values exemplify the large pressure gradient across the graft diverting flow between a high-pressure vasculature to a low-pressure vasculature. Such a large pressure gradient (> 40–50 mmHg) leads to a significant amount of shunting that results in flow reversal distal (< 0 mL/s) to the arterial anastomosis location and increased flow speeds (~ 17 cm/s proximal to shunt anastomosis to > 100 cm/s distal to shunt on both sides). The significant amount of shunting is further demonstrated by the increase in flow on the venous side distal to the graft anastomosis. Comparing the two graft configurations the SS model results in high flow rates and flow speeds. In altering the flow model to account for roughness the same quantifications do not reveal significant changes in magnitude (Table 1B and C). Pressure, velocity and volume flow rate remain nearly unchanged while the wall shear stress displays a minor upward trend for increasing roughness. It must be noted that when implementing the EB DES model with a 0.0 μm roughness (i.e., a smooth surface) the flow field solution (pressure, velocities, and WSS) becomes identical to the laminar results.

**Table 1:** Surface-averaged and time-averaged quantifications for volume flow rate (VF), pressure (P), velocity (V) and wall shear stress (WSS) at cross-sectional monitor planes shown in Fig. 2 (CP-1, CP-2, CP-3 and CP-4) for (A) laminar flow model and a smooth graft, (B) DES flow model assuming a roughness of 0.2 μm, and (C) DES flow model assuming a roughness of 1.0 μm.

Model	VF-1 (mL/s)	VF-2 (mL/s)	VF-3 (mL/s)	VF-4 (mL/s)	P-1 (mmHg)	P-2 (mmHg)	P-3 (mmHg)	P-4 (mmHg)	V-1 (cm/s)	V-2 (cm/s)	V-3 (cm/s)	V-4 (cm/s)	WSS (Pa)
(A)													
CS	3.32	− 28.60	6.66	38.62	58.58	57.72	4.84	4.70	17.01	146.56	17.38	108.11	21.06
SS	3.32	− 50.16	6.67	60.13	43.77	37.75	7.45	6.71	17.02	256.95	17.40	165.41	21.18
(B)													
CS	3.33	− 28.90	− 6.66	− 38.85	57.43	57.56	5.76	4.60	16.99	147.46	17.36	107.23	21.36
SS	3.33	− 50.52	− 6.66	− 60.49	43.80	37.42	6.46	6.10	17.00	257.83	17.37	170.68	22.10
(C)													
CS	3.33	− 28.94	− 6.66	− 38.89	57.54	57.51	5.73	4.49	16.99	147.77	17.36	107.66	21.63
SS	3.33	− 49.99	− 6.66	− 59.95	44.29	38.09	7.43	6.58	17.00	255.08	17.37	169.27	22.29

*DES* Detached Eddy Simulation.

**Figure 3:**
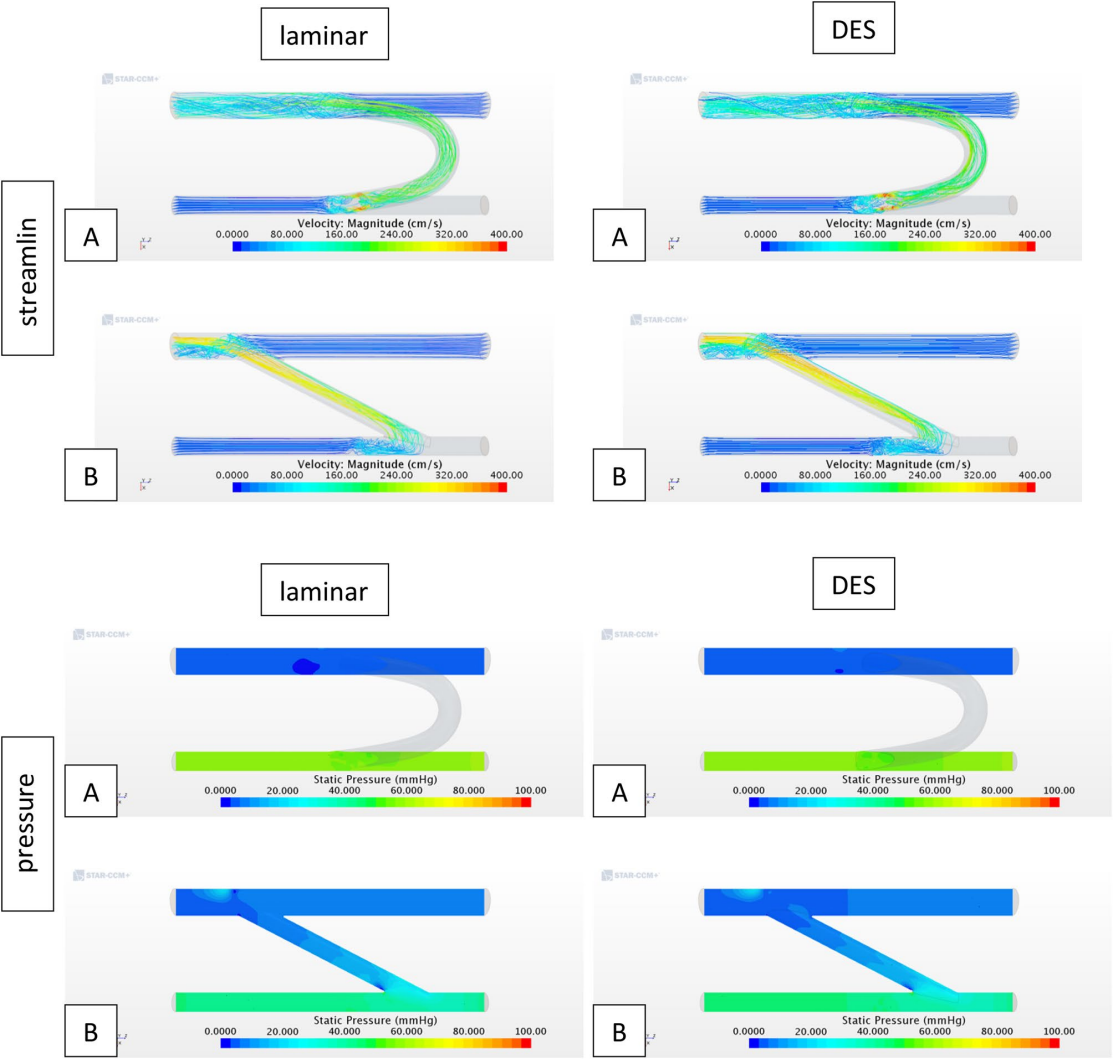
Velocity streamlines and static pressure across fluid domain for (**A**) C-shape graft and (**B**) straight graft (*DES* Detached Eddy Simulation). Static pressure contour plots exclude C-shape graft region as the graft does not lie on the same plane as the vessels.

Figure 3 offers additional insight on the flow field patterns across the domain by means of velocity streamlines. These streamlines are seeded at both inlets of the domain, hence the absence of streamlines in the distal arterial region confirm flow reversal into the shunt. While the flow in the proximal limbs of the domain present very organized and low-speed flow, in the proximity of anastomosis on the arterial side, across the graft, and distal venous anastomosis, the flow fields show some helicity, recirculation and high-speed flow. These flow features are relevant as they may be hemodynamically problematic, potentially leading to thrombogenesis. Altering the flow model has not resulted in significant flow pattern changes. Figure 3 shows the large pressure gradient across the domain between the arterial and venous sides, and once again, the pressure field for the DES model remains largely unaltered.

**Figure 4:**
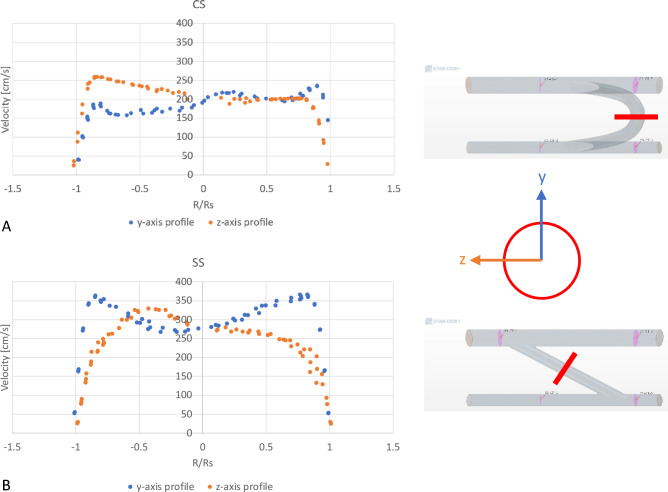
Sample velocity profiles at the mid-section of the grafts along y-axis and z-axis for (**A**) a C-shape graft and (**B**) a straight graft. The sampling cross-section is shown as a thick red line in the fluid domain (*CS* C-shaped graft, *SS* Straight graft).

Figure 4 offers additional insight regarding the velocity field across the graft. As previously reported in Tables 1A–C and Fig. 3, the straight shunt presents with significantly higher velocities along both axes. Given the shunt configuration, the shape of the velocity profiles in each model are also significantly different. In the CS model the velocity profile presents with the predictable skewed profile along the shunt curvature radial axis due to secondary or Dean flow, while along the other axis it presents nearly a top-hat profile with slight deviation. The SS model on the other hand results in less predictable near-symmetric profiles along both axes with a mild skew due to the major shunt anastomosis angle the flow must overcome as seen in Fig. 3.

### Wall shear stress

**Figure 5:**
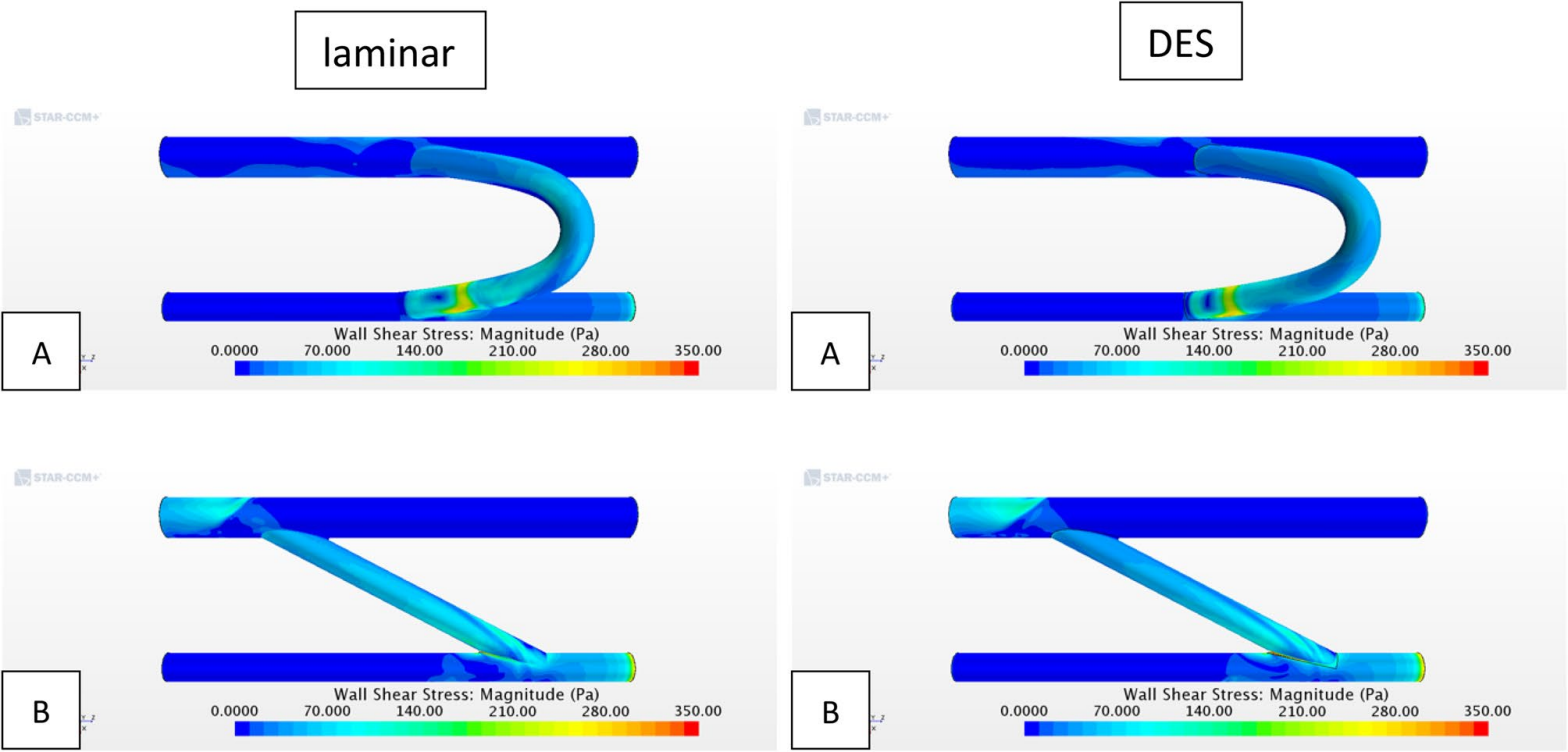
Wall shear stress across fluid domain for (**A**) C-shape graft and (**B**) straight graft (*DES* Detached Eddy Simulation).

Figure 5 supplements surface-averaged wall shear stress and velocity data in Table 1 by plotting WSS across the domain wall thereby displaying localized high shear spots which could be highly thrombogenic. As it can be seen, proximal limbs to the graft display rather low wall shear values as a result of the low flow speeds. On the other hand, across the graft and distal to the anastomosis location display far more complex WSS contours. As expected, anastomosis locations can be seen as stress concentrators, and because of the significantly larger speeds across the shunt and distal to it, WSS is increased. Updating the flow model results in noticeable alterations to the WSS distribution on the graft wall in both configurations. This is especially evident in the proximal anastomosis where the high shear spots are located (Fig. 5). Focusing on the graft, Table 2 offers further insight on the WSS mean and peak values across the shunt wall. As shown in Table 2 surface-averaged values for each configuration are similar, however, the SS graft presents with significantly elevated peak values confirming observations found in literature. When comparing Tables 1A–C, and 2 mean values are increased due to surface-averaging focusing on the shunt wall boundary where shear is observed to be higher as previously observed in Fig. 4. In accounting for roughness and turbulence the resulting mean and peak shear values have a very small increase in the CS model (~ 10 Pa), but a noticeably much larger increase in the SS model (~ 280 Pa) once again falling within the expected behavior. This outcome is due to the combined effect of graft surface roughness and the differences in velocity (or volume flow rate) magnitude in the two configurations.

**Table 2:** Time-averaged surface-average (mean) and peak wall shear stress over the graft wall region (excluding proximal and distal arteriovenous limbs) for a laminar flow model and turbulent flow model.

Model	Laminar		DES (0.2 μm)		DES (1.0 μm)	
	Mean WSS (Pa)	max WSS (Pa)	Mean WSS (Pa)	max WSS (Pa)	Mean WSS (Pa)	max WSS (Pa)
CS	52.55	364.77	52.00	374.37	52.27	364.09
SS	52.77	452.20	53.23	733.54	53.90	836.19

*CS* C-shaped graft, *DES* detached Eddy Simulation, *SS* Straight graft, *WSS* Wall shear stress.

### Power loss

Power loss ($PL$) estimations across the graft are found in Table 3 based on Eqs. (5) and (6) where $p$ is the power (in $W$), and $Q$ the volume flow rate (with $Q=v\cdot {A}_{graft}$, ${A}_{graft}$ being the graft cross-sectional area). Pressure and flow velocity are roughly sampled at a cross-sectional monitor plane 1 mm distal to the arterial anastomosis and 1 mm proximal to the venous anastomosis. For a relatively small surface roughness (0.2 μm) the SS graft displays a slightly higher power loss compared to the CS model. On the other hand, for a significantly higher surface roughness the outcome is nearly equivalent for both models. Considering the length scales of this analysis (graft diameter and surface roughness, > 5 × 10^6^ order of magnitude change), the roughness was not expected to have as a significant impact on the power loss as observed for WSS.

**Table 3:** Power loss estimation across each shunt implementation for varying graft surface roughness.

Model	DES (0.2 μm)			DES (1.0 μm)		
	${p}_{in}$ (W)	${p}_{out}$ (W)	$PL$ (W)	${p}_{in}$ (W)	${p}_{out}$ (W)	$PL$ (W)
CS	0.15	0.07	0.07	0.17	0.07	0.09
SS	0.35	0.26	0.09	0.35	0.26	0.08

*CS* C-shaped graft, *DES* Detached Eddy Simulation, *PL* power loss, *SS* straight graft.

5 $$p=\left(P+\frac{1}{2}\rho {v}^{2}\right)Q$$

6 $$PL={p}_{in}-{p}_{out}$$

Table 3 also reveals how the CS model presents with lower overall flow power (0.15–0.17 W) at the inlet compared to the straight SS model (~ 0.35 W), which then results in significantly higher flow power introduced in the venous side in the SS model. This outcome may become problematic and lead to platelet activation and increased intimal cell proliferation.

## Discussion

Improving our understanding of the best configuration for a vascular arteriovenous shunt is of paramount importance in improving the patency and longevity of a shunt graft. In this study we evaluated the configuration of a novel silicon-lined vascular shunt as well as its surface finishing using computational fluid dynamic modeling which has been proven to be a reliable method to evaluate the flow pattern and shear forces across a shunt^23^. This has allowed us to identify the best anastomotic and geometric configuration as well as the effect of the surface roughness on shear forces and pattern of blood flow which can affect the longevity and patency of the shunt leading to shunt failure. This study was intended to be an in-silico study prior to moving forward with an animal model evaluation.

The study confirms previous work performed by Kuwahara et al. that a C-shaped shunt performs better than a straight shunt with less shear forces and better flow pattern potentially reducing thrombus formation through platelet activation^24^ and neointimal growth that usually occurs at the arterial and venous anastomotic sites^25^. Additionally, we showed that the energy transferred to the fluid mass from the arterial side is significantly higher in the straight shunt. This energy needs to dissipate as the fluid travels through the shunt leading to increase shear forces on the wall. The transfer to the relatively low energy fluid on the venous side exerts higher shear forces on the venous anastomosis, a factor that can lead to platelet activation and neointimal growth^26^.

The choice for silicon as a lining for the PTFE conduit was based on previous work done by Boggs et al. Their work shows a significantly lower platelet adhesion to surface-finished silicon, using magnetic abrasive technique achieving sub micrometer peak to valley surface roughness with decreased thrombogenicity^27^. While the unlined surface, without silicon, shows higher platelet adhesion and activation^28^.

Furthermore, our data suggests that the smoother finishing of the surface also appears to be a factor in potentially reducing the shear forces and associated platelet activation^24^. Although the power loss in the smoother surface C-shaped shunt only dropped by about 22%, we believe that this will be an important factor once applied in-vivo.

The combination of the decreased adhesiveness to a mechanically finished silicone surface and the marginally lower shear force shown in our data is expected to lower the impact on the platelets decreasing the degree of change incurred by the platelets leading to activation of the clotting cascade. The first step in the activation process is the formation of filopods that is generated by the shear force. The platelets still roll having this shape. However, the activation will start after the platelet firmly adheres to the surface of the conduit releasing factors that will promote recruitment of further platelets and release factors that will activate the clotting cascade. We hypothesize that the smooth surface will decrease the irreversible adhesion of platelets to the surface and that curtails the starting of the clotting process^24, 27^.

The ideal conduit for vascular reconstruction continues to be the primary interest of researchers and clinicians in cardiovascular surgery. Despite significant improvements over the years, the use of autologous, tissue engineered, or prosthetic material continues to harbor limitations including durability, thrombogenicity, and off-the-shelf availability. In this study, after evaluating the CFD parameters involving a straight versus C-shaped conduit for an arteriovenous shunt, we established that the shear forces and flow patterns were superior for a curved conduit. The risk of thrombosis and its mechanism in a high shear stress environment have been documented by Lazo-Langner et al.^26^. In addition, our data showed a medical grade silicon lined graft with smoother surface finishing, and peak to valley roughness of 200 nm or less, had lower power loss across the shunt.

### Future direction

As such, our aim is to trial the implantation of a 5 mm C-shaped arteriovenous novel silicon-lined shunt from the internal carotid artery to the external jugular vein in the sheep model. The CFD model generated in this study is based on our planned animal model neck anastomosis to be performed in a beveled end-to-side fashion. This technique can enlarge the anastomosis and potentially mitigate thrombosis and neointimal growth^29^.

In addition, external ring reinforced support will be used to prevent kinking of the shunt. Optimal microvascular and fine suture techniques will be used to minimize anastomotic scarring and neointimal proliferation. This graft will be imaged in vivo and subsequently explanted to evaluate factors such as hemodynamics, patency, intimal hyperplasia, and thrombogenicity. The generated data will assist in planning the future course of our studies of a prosthetic silicon-lined vascular conduit. To address limitations of this study related to the absence of significant pulsatiliy, especially on the arterial side and the nature of the imposed boundary conditions, ongoing analyses are implementing a detailed lumped parameter model (LPM) that will generate high fidelity time-dependent boundary conditions. This LPM will be loosely-coupled to the CFD domain to first iteratively converge the boundary conditions and then collect hemodynamic measurements.

### Limitations

This study does not account for in vivo factors including individual hemodynamic changes associated with age, blood pressure, cardiac output, rate of neointimal growth, and thrombogenic profile. For this reason, further evaluation using an animal model is required for proof of concept prior to moving to clinical trials. We also did not account for vascular pulsatility and compliance which can affect the shear forces and thereafter the shunt integrity.

The CFD modeling is limited by the rigid-wall assumption which may cause an overestimation of the shear stresses calculated. As previously mentioned, the boundary conditions imposed do not account for pulsatility and no coupling to a lumped parameter model was performed. In addition, in order to account for roughness, the modeling approach used (DES), implements roughness numerically as opposed to a high degree mesh refinement to physically account for surface features. We plan to perform detailed surface feature evaluation in a follow up study.

### Supplementary Information

Supplementary Information.

## Data Availability

The datasets used and/or analyzed during the current study available form the corresponding author on reasonable request.
