# Supplementary material for: Preliminary in-silico analysis of vascular graft implantation configuration and surface modification
Source: Sci Rep. 2023 Oct 2;13:16539. doi: 10.1038/s41598-023-42998-y (PMC10545661; doi:10.1038/s41598-023-42998-y)
Supplement: Supplementary file 1 — Supplementary Information. [file 41598_2023_42998_MOESM1_ESM.docx]

Supplementary Material


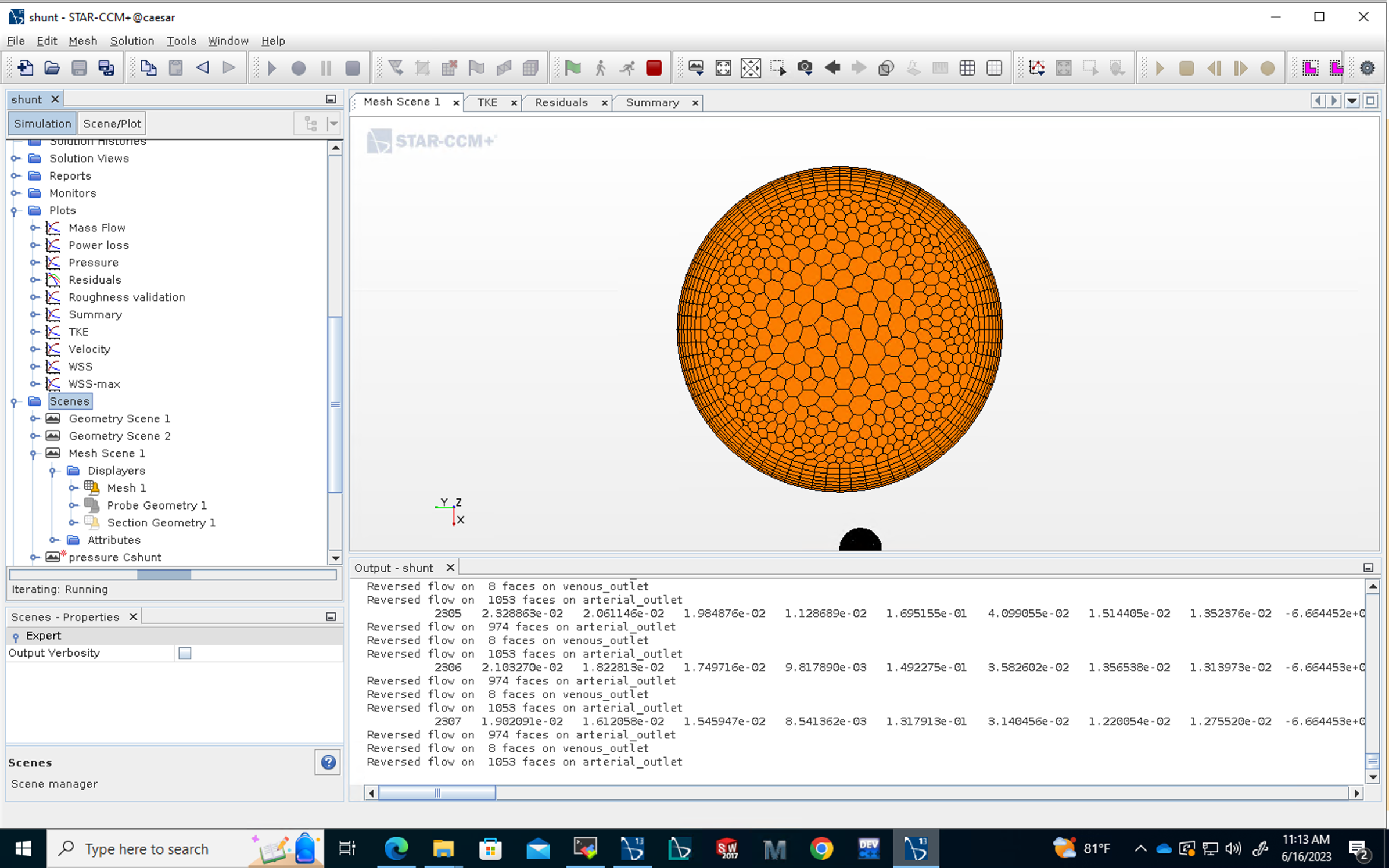


Figure S1 – Sample boundary mesh following grid independence study implementing unstructured polyhedral cells with 5 wall boundary prism layers.

Table S1 – Grid independence study for the C-shape graft sampling surface-averaged wall shear stress (WSS) averaged over the entire domain wall, time-averaged static pressure (P-1, P-2, P-3, and P-4) and velocity (V-1, V-2, V-3 and V-4) measurements are surface-averaged and time-averaged at four probe planes across the fluid domain shown in Figure 2-A CP-1, CP-2, CP-3 and CP-4). GCI (grid convergence index), order of convergence and ROC (range of convergence) were estimated based on the methodology reported in literature [20, 21], where ${GCI}_{i,i+1}=F_{s}\frac{\left| f_{i}-f_{i+1} \right|}{f_{i}\left( r^{p}-1 \right)}$ ($f$ a scalar value and $F_{s}$ a safety factor, typically 1.25) with $p=\frac{ln\left( \frac{f_{i}-f_{i+1}}{f_{i+1}-f_{i+2}} \right)}{ln\left( r \right)}$ (order of convergence), $r=\frac{\Delta_{i}}{\Delta_{i+1}}$ (refinement ratio) and $ROC=\frac{{GCI}_{i,i+1}}{{GCI}_{i+1,i+2}r^{p}}\approx1$. When ROC$\approx$1 the grid can be considered within the range of asymptotic convergence. To maintain a consistent grid refinement ratio $r$, cases 1, 3 and 5 were selected to carry out the grid independence study.

| Case # | base size (mm) | mesh count | CS-P-1 (mmHg) | CS-P-2 (mmHg) | CS-P-3 (mmHg) | CS-P-4 (mmHg) | CS-V-1 (cm/s) | CS-V-2 (cm/s) | CS-V-3 (cm/s) | CS-V-4 (cm/s) | CS-WSS (Pa) |
| --- | --- | --- | --- | --- | --- | --- | --- | --- | --- | --- | --- |
| 1 | 2.00 | 208511 | 57.25 | 58.93 | 6.09 | 5.13 | 17.10 | 145.74 | 17.42 | 101.49 | 20.81 |
| 2 | 1.50 | 374485 | 57.40 | 58.77 | 5.74 | 5.06 | 17.05 | 146.91 | 17.38 | 102.47 | 21.00 |
| 3 | 1.00 | 763134 | 57.47 | 58.87 | 5.78 | 5.02 | 16.99 | 146.19 | 17.36 | 102.98 | 21.47 |
| 4 | 0.75 | 1189759 | 57.13 | 58.89 | 5.69 | 4.99 | 17.00 | 146.01 | 17.36 | 103.12 | 21.54 |
| 5 | 0.50 | 1415692 | 57.56 | 58.84 | 5.63 | 4.94 | 17.00 | 146.40 | 17.34 | 103.17 | 21.72 |
| p | - | - | 1.28 | 1.46 | 1.01 | 0.67 | - | 1.17 | 1.42 | 2.96 | 1.43 |
| GCI_13_ | - | - | 0.33 | 0.08 | 6.24 | 4.91 | - | 0.31 | 0.23 | 0.27 | 2.37 |
| GCI_35_ | - | - | 0.14 | 0.03 | 3.27 | 3.16 | - | 0.14 | 0.09 | 0.03 | 0.85 |
| ROC | - | - | 1.00 | 1.00 | 0.95 | 0.98 | - | 1.00 | 1.00 | 1.01 | 1.03 |

Table S2 – Grid independence study for the S-shape graft sampling surface-averaged wall shear stress (WSS) averaged over the entire domain wall, time-averaged static pressure (P-1, P-2, P-3, and P-4) and velocity (V-1, V-2, V-3 and V-4) measurements are surface-averaged and time-averaged at four probe planes across the fluid domain shown in Figure 2-B CP-1, CP-2, CP-3 and CP-4). GCI (grid convergence index), order of convergence and ROC (range of convergence)were estimated based on the methodology reported in literature [20, 21], where ${GCI}_{i,i+1}=F_{s}\frac{\left| f_{i}-f_{i+1} \right|}{f_{i}\left( r^{p}-1 \right)}$ ($f$ a scalar value and $F_{s}$ a safety factor, typically 1.25) with $p=\frac{ln\left( \frac{f_{i}-f_{i+1}}{f_{i+1}-f_{i+2}} \right)}{ln\left( r \right)}$ (order of convergence), $r=\frac{\Delta_{i}}{\Delta_{i+1}}$ (refinement ratio) and $ROC=\frac{{GCI}_{i,i+1}}{{GCI}_{i+1,i+2}r^{p}}\approx1$. When ROC$\approx$1 the grid can be considered within the range of asymptotic convergence. To maintain a consistent grid refinement ratio $r$, cases 1, 3 and 5 were selected to carry out the grid independence study.

| Case # | base size (mm) | mesh count | SS-P-1 (mmHg) | SS-P-2 (mmHg) | SS-P-3 (mmHg) | SS-P-4 (mmHg) | SS-V-1 (cm/s) | SS-V-2 (cm/s) | SS-V-3 (cm/s) | SS-V-4 (cm/s) | SS-WSS (Pa) |
| --- | --- | --- | --- | --- | --- | --- | --- | --- | --- | --- | --- |
| 1 | 2.00 | 197828 | 44.35 | 38.51 | 6.88 | 6.38 | 17.16 | 253.28 | 17.42 | 163.62 | 20.90 |
| 2 | 1.50 | 340993 | 43.53 | 38.01 | 7.51 | 6.55 | 17.08 | 255.45 | 17.38 | 163.08 | 21.10 |
| 3 | 1.00 | 716337 | 44.12 | 38.20 | 7.64 | 6.77 | 17.00 | 254.41 | 17.37 | 166.68 | 21.97 |
| 4 | 0.75 | 1054902 | 44.43 | 38.19 | 7.44 | 6.65 | 17.00 | 254.61 | 17.37 | 168.29 | 22.56 |
| 5 | 0.50 | 1370899 | 44.05 | 38.08 | 7.64 | 6.98 | 17.00 | 255.06 | 17.37 | 168.05 | 22.57 |
| p | - | - | 1.75 | 1.48 | 6.62 | 0.90 | - | 0.80 | 4.15 | 1.17 | 0.85 |
| GCI_13_ | - | - | 0.28 | 0.58 | 0.14 | 8.92 | - | 0.75 | 0.02 | 1.88 | 8.05 |
| GCI_35_ | - | - | 0.08 | 0.21 | 0.00 | 4.51 | - | 0.43 | 0.00 | 0.82 | 4.26 |
| ROC | - | - | 0.99 | 0.99 | 1.11 | 1.06 | - | 1.00 | 1.00 | 1.02 | 1.05 |

Table S3 - Nondimensional roughness parameter and cell height estimations for the EB DES model and varying surface roughness.

| Roughness | CS-$R^{+}$ | CS-$y^{+}$ | SS-$R^{+}$ | SS-$y^{+}$ |
| --- | --- | --- | --- | --- |
| 0.2um | 0.01 | 1.79 | 0.01 | 1.86 |
| 1.0um | 0.05 | 1.79 | 0.06 | 1.87 |
